# Cadmium and its inorganic compounds – Addendum: re-evaluation of the BLW: Assessment Values in Biological Material – Translation of the German version from 2025

**DOI:** 10.34865/bb744043e10_4ad

**Published:** 2025-12-22

**Authors:** Ernst Hallier, Hans Drexler, Andrea Hartwig, MAK Commission

**Affiliations:** 1 37136 Ebergötzen Germany; 2 Friedrich-Alexander-Universität Erlangen-Nürnberg. Institute and Outpatient Clinic of Occupational, Social, and Environmental Medicine Henkestraße 9–11 91054 Erlangen Germany; 3 Institute of Applied Biosciences. Department of Food Chemistry and Toxicology. Karlsruhe Institute of Technology (KIT) Adenauerring 20a, Building 50.41 76131 Karlsruhe Germany; 4 Permanent Senate Commission for the Investigation of Health Hazards of Chemical Compounds in the Work Area. Deutsche Forschungsgemeinschaft, Kennedyallee 40, 53175 Bonn, Germany. Further information: Permanent Senate Commission for the Investigation of Health Hazards of Chemical Compounds in the Work Area | DFG

**Keywords:** cadmium, biological guidance value, BLW, nephrotoxicity, Urin, urine

## Abstract

The German Senate Commission for the Investigation of Health Hazards of Chemical Compounds in the Work Area (MAK Commission) re-evaluated the data for cadmium [7440-43-9] to derive a biological guidance value (BLW) for its systemic non-carcinogenic end points. Relevant studies were identified from a literature search. Exposure to cadmium dust can cause nasal inflammation and anosmia, bronchitis and pneumonia, which are recognised as local effects. Tubular kidney damage was identified as the most sensitive end point of systemic toxicity, which results in excretion of low molecular weight proteins such as α1 and β2-microglobulins and retinol binding protein (RBP) in the urine. Recent studies on workers occupationally exposed to cadmium revealed a NOEL (no observed effect level) and a BMDL5 (benchmark dose lower limit) for tubular proteinuria at approximately 3 to 5 µg cadmium/g creatinine in ever-smokers, whereas this threshold is higher in never-smokers. Therefore, a BLW of 2 µg cadmium/g creatinine is set for cadmium in urine.

**Table:** 

**BLW (2024)**	**2 µg cadmium/g creatinine**
**BAR (2010)**	**1 µg cadmium/l blood** ^a)^ **0.8 µg cadmium/l urine** ^a)^
	Sampling time: not fixed in the steady state
	
**MAK value**	**–**
Absorption through the skin (2004)	H
Carcinogenicity (2004)	Category 1

^a)^ other values apply for smokers

## Re-evaluation

In 2004, cadmium was classified in Carcinogen Category 1 (translated in Greim 2006). This was mainly due to lung tumours caused by inhalation of cadmium compounds, and tumour findings in the kidneys were also reported. A concentration at which no carcinogenic effect was observed could not be determined. A biological tolerance value (BAT value) can therefore not be derived. Exposure to cadmium dust and vapours causes acute and subchronic inflammation of the respiratory tract and lungs (“cadmium rhinitis” and anosmia, bronchitis and pneumonia), which can primarily be described as local effects.

Regarding systemic effects of cadmium, the kidneys are the main target organ. The kidneys accumulate cadmium during chronic exposure and are therefore both the main storage organ and a critically endangered target organ. In particular, the renal tubules and, at higher levels of exposure, the glomeruli are also damaged. Due to the impaired reabsorption in the renal tubules, more calcium and phosphate are excreted in the urine. This can lead to osteomalacia with spontaneous fractures of the bones (described in the 1950s as “Itai-Itai disease”) and to the formation of urinary stones. Additional osteoporosis with loss of bone matrix (reduced bone density) has been reported. Women are particularly at risk of bone damage from cadmium, among other things due to postmenopausal osteoporosis, increased calcium loss through breastfeeding, reduced metallothionein protein production and iron deficiency, which causes increased intestinal absorption of cadmium.

Work-related exposure to cadmium and its inorganic compounds occurred in earlier decades during the coating of metals for corrosion protection (cadmium plating) and the processing of such coated metals (welding, separating, cutting). The largest quantitative use was in the manufacture of nickel-cadmium accumulators and batteries, especially rechargeable batteries, which accounted for more than 80% of the global use of cadmium in the 1990s and 2000s. Cadmium compounds are also used for pigments in the photographic industry and photovoltaics, in the manufacture of special glass (e. g. television screens, traffic lights) and as stabilisers for plastics. As cadmium has now been replaced by other metals in battery production, the industrial significance of this metal and its compounds has declined considerably. Today, occupational hazards are primarily associated with the disposal and recycling of old batteries.

In 2007, a biological guidance value (BLW) of 7 µg cadmium/l urine was derived for cadmium. This was based on the nephrotoxicity of cadmium observed during occupational exposure. At that time, it was concluded that above a concentration of 5 µg cadmium/g creatinine (corresponding to 7.5 µg cadmium/l urine), tubular kidney damage occurred (translated in Käfferlein et al. 2016). In addition, it was assumed that proteinuria was reversible at levels below 5 µg cadmium/g creatinine or below 7.5 µg/l urine.

In 2010, the BLW of 7 µg cadmium/l urine was withdrawn (translated in Drexler et al. 2016). This withdrawal was based on indications that tubular proteinuria is irreversible and that even a lower burden of 4 µg cadmium/g creatinine can lead to glomerular damage with a reduced glomerular filtration rate (GFR) (Järup et al. 1995). In addition, studies on environmental exposures indicated that a reduced GFR can occur at a similarly low cadmium burden as that observed in the case of tubular damage (Åkesson et al. 2005; Suwazono et al. 2006), and that the probability of conspicuously elevated values for tubular microproteins is 10% at an excretion of 1–2 µg cadmium/g creatinine (Buchet et al. 1990, 1991). Also the long half-life with the accumulation of cadmium in the kidneys is to be taken into account.

Since the last evaluation of the BLW in 2010, further studies on occupationally and environmentally exposed people have been published, but above all new findings on the pathophysiology of the kidneys have been published that make a re-evaluation necessary.

## Pathophysiological aspects of the nephrotoxicity of cadmium

### Metallothionein

The classic hypothesis on cadmium toxicity in the kidneys, which has been proven by numerous research studies in vivo and in vitro, states that cadmium is bound to the protein metallothionein. This low-molecular protein is produced mainly in the liver, but also to a lesser extent in renal tubule cells (Klaassen et al. 2009). After glomerular filtration, it enters the primary urine, is reabsorbed in the proximal tubule and accumulates there. Over time, the metallothionein degenerates so that cadmium is released again and causes local toxicity. This results in tubular damage to the kidney, and, as a result of the impaired physiological reabsorption, the excretion of electrolytes (including calcium, phosphate) and low-molecular proteins such as α1-microglobulin (α1MG), β2-microglobulin (β2MG), retinol-binding protein (RBP) and *N*-acetyl-β-D-glucosaminidase (NAG) in the final urine is increased. These microproteins are analysed in the laboratory as markers of tubulopathy. The upper normal limit for β2MG in spontaneous urine given in the German clinical literature is 0.3 mg/l or 0.2 to 0.3 mg/g creatinine (Gressner and Arndt 2019). The continuous release of cadmium from metallothionein and its concentration in the final urine correlates with the total burden of cadmium stored in the renal cortex. The cadmium concentration in urine is therefore regarded as a measure of chronic cadmium exposure.

This hypothesis applies to healthy adults of typical working age. However, the situation is problematic in the case of glomerular or interstitial kidney damage, e. g. diabetic nephropathy or chronic glomerulonephritis. Metallothionein has a high affinity for cadmium, but the metal also binds less specifically to various other proteins (Fels et al. 2019), especially if total protein excretion (albumin) is increased due to glomerulopathy. In addition, cadmium homeostasis is dependent on other metals competing for protein binding, particularly the iron (Thévenod and Wolff 2016) and zinc balance (Nordberg and Nordberg 2022).

### Age and creatinine adjustment

One aspect to consider when deriving a BLW is age, as the lower creatinine excretion of people over 60 or 70 years, possibly with chronic diseases, inevitably leads to a statistical association between higher creatinine-adjusted cadmium concentrations and (pathological) proteinuria. However, this association is not due to cadmium causing old age or chronic diseases, but rather to the fact that an adjusted high cadmium concentration results from reduced creatinine excretion. Satarug et al. (2021) also pointed out the problem of creatinine adjustment and called for the creatinine clearance to be determined. Based on their study data, they demonstrated that the effect of cadmium on GFR is reversed by normalizing the excretion rate via creatinine clearance.

The inclusion of adolescents is also problematic, particularly in population-based environmental epidemiological studies. In terms of volume, the cadmium concentration in urine drops to a minimum of approximately 0.2 µg/l in 20-year-olds and then rises to a maximum between the ages of 60 and 70 years (Chaumont et al. 2013). Related to creatinine, however, children and adolescents have urinary cadmium concentrations that are similar to those or even higher than those in adults, although their body burden of the metal is 5 to 10 times lower (Bernard 2016).

### Reverse causation

In the case of non-occupational health problems affecting kidney function and protein excretion, the dose-response relationship between cadmium concentration and the excretion of tubular marker proteins may be distorted. This phenomenon has been described by various authors as “reverse causation”, which acts as a confounding factor, particularly at low cadmium exposure levels (Åkesson et al. 2014; Bernard 2016; Buser et al. 2016; Vacchi-Suzzi et al. 2016 a, b).

The positive association between the excretion of low molecular weight proteins and the cadmium concentration in urine is weaker in never smokers than in smokers (Åkesson et al. 2014). Bernard (2016) pointed out that in environmental studies, cadmium is regarded to cause disorders, including growth retardation, impaired child development, bone demineralization and fractures, kidney dysfunction and disease, reproductive impairment, diabetes, hypertension etc. even at cadmium concentrations of < 0.5 µg/g creatinine. This would imply that cadmium is more toxic at low exposure levels than at high exposure levels (Bernard 2016). However, kidney donors with a median cadmium concentration in urine of 12.9 µg cadmium/g creatinine showed no impairment of glomerular function (Wallin et al. 2016).

Buser et al. (2016) reported on the results of the population-based US National Health and Nutrition Examination Survey (NHANES) regarding the relationship between cadmium and lead levels in blood and urine and kidney function. NHANES described an inverse association between GFR and cadmium in blood, with a positive association between GFR and albumin in urine and cadmium concentration in urine at low cadmium exposure levels. Reverse causation suggests that reduced GFR lowers the overall filtration of chemical substances, leading to reduced levels in urine and increased levels in blood. Decreased glomerular filtration thus causes an increase in cadmium in blood with a simultaneous reduction in cadmium excretion in urine.

Byber et al. (2015) conducted a systematic review of the association between cadmium or cadmium compounds and kidney disease in occupationally exposed workers and in the general population. Studies with 34 exposed groups, totalling more than 3000 participants, were evaluated. The systematic evaluation found no evidence supporting a risk of progression from primary tubular kidney damage caused by cadmium to chronic kidney disease (reduced GFR).

### Reversibility

As already reported by Drexler et al. (2016), Järup et al. (1995) investigated the relationship between cadmium exposure and the glomerular filtration rate (GFR) in 42 solderers with at least five years of occupational cadmium exposure. Twenty-four workers exhibited tubular proteinuria with β2MG excretion of > 34 µg/mmol creatinine (> 304 µg/g creatinine) and 17 workers had β2MG excretion of > 60 µg/mmol creatinine (> 536 µg/g creatinine). In 12 workers whose GFR had been measured three times during the study (1984, 1989 and 1993), no improvement in GFR was observed even years after the end of occupational exposure.

Roels et al. (1997) investigated the development of cadmium-induced renal tubular dysfunction in workers according to the severity of microproteinuria in 32 workers with a history of high cadmium exposure during two observation periods (1980–1984 and 1990–1992). β2MG concentrations in urine not exceeding 300 μg/g creatinine were associated with a low risk of subsequently developing tubular dysfunction, even in cases with historical cadmium concentrations in urine occasionally > 10 but always < 20 μg/g creatinine. There was evidence of a reversible tubulotoxic effect of cadmium when mild microproteinuria was present (β2MG in urine > 300 but < 1500 μg/g creatinine) and cadmium concentrations in urine never exceeded 20 μg/g creatinine. In cases of severe microproteinuria (β2MG in urine > 1500 μg/g creatinine) and historical cadmium concentrations in urine exceeding 20 μg/g creatinine, cadmium-induced tubular dysfunction was progressive despite reduction or cessation of cadmium exposure.

Trzcinka-Ochocka et al. (2002) examined 58 employees of a nickel-cadmium battery factory who had not been exposed to cadmium for at least one year and who had been exposed to geometric mean levels of 23.3 and 55.7 µg cadmium/l blood in 1983 and 1986–1988, respectively. The working group wanted to assess the reversibility of renal tubular dysfunction in relation to the severity of microproteinuria, cadmium concentration in urine and time since the end of exposure. The employees were divided into three groups according to their RBP concentrations in urine in 1986–1988: (i) < 300 (n = 26), (ii) 301–1501 (n = 25) and (iii) > 1501 µg/g creatinine (n = 7). In 1999, RBP levels were below 300 µg/g creatinine in 85% (i), 64% (ii) and 42% (iii) of the persons. There were indications that even in cases of relatively high previous exposure to cadmium, tubular proteinuria and possibly also the decline in the glomerular filtration rate may be reversible.

In the longitudinal study by Gao et al. (2016) investigating 41 non-smoking female workers at a factory manufacturing nickel-cadmium batteries, the median follow-up period after the end of exposure was 8 years (1–10 years). Using a regression model, a decrease in the cadmium concentration in urine of 3.0 µg/g creatinine on average over 10 years was estimated. The concentrations of β2MG and RBP fluctuated relative to their respective baseline concentrations.

## Relationship between internal exposure and effects

### Occupational exposure

Ding et al. (2011) examined 103 railway welders (19 ♂, 84 ♀) in China with an occupational exposure duration of 2 to 21 years (12.8 ± 7.27). Cadmium concentrations in the workplace air were in the range from 8 to 86 µg/m^3^ and 17% of measured data exceeded the limit value of 10 µg/m^3^. The study does not provide any information on smoking habits. Cadmium concentrations in urine were in the range from 0.05 to 12.4 µg/g creatinine. Welders with more than 3 µg cadmium/g creatinine had statistically significantly higher β2MG values than those with less than 3 µg/g creatinine. The publication probably gives mean values and standard deviations in Table 1, but precise information on this is lacking. The implausible standard deviations are striking. The two highest β2MG concentrations were 920 and 935 µg/g creatinine. The authors concluded that exposure to welding fumes can lead to elevated levels of cadmium in urine and markers of tubular damage. No other clinical examinations were performed on the welders in the study.

A Belgian working group examined 184 healthy male workers at a zinc smelter (n = 132) and 52 employees at a factory producing acrylic fibre blankets in Algeria (Haddam et al. 2011). The median cadmium concentrations in the blood of the zinc smelter workers were 0.80 µg/l (interquartile range (IQR 0.45–1.16) and in the urine 0.70 µg/g creatinine (IQR 0.40–1.3), while the median cadmium concentrations in the blood of the blanket factory employees were 0.66 µg/l (IQR 0.47–0.87) and in the urine 0.55 µg/g creatinine (IQR 0.40–0.90). The 55 smoking workers at the zinc smelter had statistically significantly higher cadmium concentrations in their blood (median 1.20 µg/l; IQR 0.80–1.70) and urine (median 1.00 µg/g creatinine; IQR 0.70–1.37) and higher excretion of retinol-binding protein (RBP) and protein HC (α1-microglobulin) in urine. However, RBP in urine correlated statistically significantly with cadmium concentrations in urine (r = 0.27; p = 0.04) and blood (r = 0.29; p = 0.02) only in never-smokers. These correlations were not observed in ex-smokers (urine: r = 0.21; p = 0.33 and blood: r = 0.06; p = 0.15) and smokers (urine: r = –0.04; p = 0.74 and blood: r = 0.02; p = 0.87). This led the authors to conclude that the higher RBP excretion in urine associated with smoking cannot be explained by higher cadmium exposure and that smoking has a stronger influence on cadmium concentrations in urine than occupation. Overall, cadmium concentrations in blood and urine were within the range of background exposure.

A Belgian working group investigated a cohort of 599 employees (451 ♂, 148 ♀) with a mean age of 45.4 years who worked in four nickel-cadmium battery factories in France, Sweden and the USA for an average period of 18.8 years (Chaumont et al. 2011). The study investigated whether the relationship between urinary protein excretion and urinary cadmium concentration was influenced by gender, age, diuresis, and smoking. The employees were categorised into 7 exposure groups based on their urinary cadmium concentrations (≤ 1, > 1–2, > 2–3, > 3–4, > 4–6, > 6–10, > 10 µg cadmium/g creatinine). Workers with less than 1 µg cadmium/g creatinine served as the reference group. Including all employees, the odds for “abnormal” (> 95^th^ percentile of the reference group) concentrations of RBP and β2MG in urine were statistically significantly increased in the groups with cadmium concentrations in urine of 6 to 10 µg/g creatinine and more than 10 µg/g creatinine. The benchmark dose (BMD5) and benchmark dose lower limit (BMDL5) for a 5% increase in the background prevalence of “abnormal” concentrations of RBP and β2MP in urine were estimated to be 5.1/3.0 µg cadmium/g creatinine and 9.6/5.9 µg cadmium/g creatinine, respectively. When ever-smokers were excluded, the levels of both proteins in urine were elevated only in workers with a cadmium concentration in urine of more than 10 µg/g creatinine (odds ratios 21.8; 95% CI 6.4–74.4 and 15.1; 3.6–63.1, respectively). For never smokers, the BMD5/BMDL5 of cadmium in urine were 12.6/6.6 and 12.2/5.5 µg/g creatinine, respectively, while for ever-smokers they were 6.2/4.9 and 4.3/3.5 µg/g creatinine, respectively. The authors derived a benchmark dose lower limit (BMDL5) of cadmium in urine for low molecular weight proteinuria resulting from occupational exposure to cadmium of 5.5 to 6.6 µg/g creatinine. The authors concluded that tobacco smoking damages kidney function even in people without high blood pressure or diabetes. A reliable determination of the benchmark dose could only be made if ever-smokers were excluded. Confounding of the associations between low molecular weight proteins and cadmium concentration in urine appears to stem primarily from the renal damage associated with chronic smoking.

Gao et al. (2016) conducted a longitudinal study in Shenzhen (China) on 41 non-smoking female workers at a nickel-cadmium battery manufacturing factory at the time of termination of cadmium exposure and for up to 10 years after the end of exposure. At the end of exposure, the mean age was 30.8 ± 6.7 years, and the duration of employment was 7.5 ± 4.2 years; urinary concentrations were determined for cadmium (median 6.19 µg/g creatinine; IQR 5.17–7.45), β2MG (median 105.38 µg/g creatinine; IQR 76.16–190.23) and RBP (median 71.84 µg/g creatinine; IQR 44.98–115.57). Cadmium concentrations correlated in a statistically significant manner with β2MG and RBP concentrations. The authors thus confirmed the known association between chronic cadmium exposure and renal tubular function. Neither age nor length of employment had a significant influence on the concentrations of cadmium, β2MG or RBP.

Choi et al. (2020) examined 10 workers of a small-scale silver soldering company in South Korea who had been employed/exposed for an average of 8.5 years (3–20 years). Cadmium concentrations in the air were in the range from 6 to 15 µg/m^3^. In all 10 workers, blood cadmium concentrations averaged 21.29 µg/l (maximum 34.6 µg/l). In 9 workers, cadmium concentrations in urine averaged 22.15 µg/g creatinine (maximum 62.9 µg/g creatinine). The β2MG concentration was elevated in 3 workers. Urinary cadmium concentration was positively associated with urine protein concentration. The authors concluded that cadmium intoxication may occur at quite low air concentrations. In the graphical representation of the linear correlation of the values, 30 µg cadmium/g creatinine corresponded to approximately 0.2 mg β2MG/l urine.

### Environmental health studies

Whereas occupational exposure to cadmium is almost exclusively by inhalation, non-occupational exposure primarily involves the inhalation of tobacco smoke and, above all, oral dietary intake. The following section describes environmental health studies on cadmium published since 2010 with the end point of kidney toxicity:

Ferraro et al. (2010) evaluated data from the US National Health and Nutrition Examination Survey (NHANES). In 5426 subjects (48.7% ♂, 51.3% ♀, mean age 47 years), including 48.8% smokers, cadmium concentrations in blood and urine were associated with a higher proportion of chronic kidney disease (GFR < 60 ml/min/1.73 m^2^) and albumin excretion in urine. The results were adjusted for age, gender, body mass index and ethnicity. In subjects with cadmium concentrations > 1 µg/g creatinine in urine and subjects with > 1 µg/l in blood, a higher association with albuminuria (OR 1.41) and chronic kidney disease was found.

Suwazono et al. (2011) conducted a population-based study of persons aged 50 years and older (547 ♂, 723 ♀) living in a non-cadmium-contaminated area of Japan. The BMDL5 for β2MG was 2.6 µg cadmium/g creatinine in men and 1.4 µg cadmium/g creatinine in women, and for NAG it was 4.1 µg cadmium/g creatinine in men and 3.1 µg cadmium/g creatinine in women. No data for smoking habits are available.

Akerstrom et al. (2013) examined 24-hour urine samples from 30 healthy non-smokers of both sexes in Sweden, with a median age of 39 years. The cadmium concentration in urine was positively associated with the excretion of albumin and α1MG. Albuminuria occurred at cadmium concentrations above 2 µg/l urine or 2.5 µg/g creatinine, and increased α1MG excretion occurred above a cadmium concentration of 3 µg/l or 2.9 µg/g creatinine. Intraindividually, the associations were stronger for excretion rates and concentrations adjusted for specific gravity than for concentrations adjusted for creatinine. The authors concluded that in non-smokers, associations between cadmium in urine and markers of kidney function were not due to cadmium toxicity but result from normal variation in renal function.

Thomas et al. (2013) examined patients (599 ♂, 253 ♀) in England and Sweden with chronic kidney disease (GFR < 60 ml/min). No association was found between chronic kidney disease and dietary cadmium exposure (median 0.219 µg/g creatinine (0.22 nmol cadmium/mmol creatinine)).

Hu et al. (2014) investigated the relationship between urinary cadmium and the tubular protein markers β2MG and NAG in urine in 490 non-smoking women aged 35 to 54 years from two counties in China. With increasing cadmium concentrations, the tubular proteins NAG and β2MG in urine increased significantly. For the 90^th^ percentile as cut-off criterion, the BMD5/BMDL5 for these effects were 2.08/1.41 and 3.80/2.18 µg cadmium/g creatinine, respectively, in the first county and 3.34/1.91 and 0.99/0.74 µg cadmium/g creatinine, respectively, in the second county. The authors concluded that the BMD5 for cadmium in urine in China was similar to the reference value of 1 µg cadmium/g creatinine specified by the European Food Safety Authority.

Ke et al. (2015 a, b) conducted a cross-sectional study of 6103 subjects aged 35–89 years (2715 ♂, mean age 60.3 years; 3388 ♀, mean age 59.5 years) from five Chinese provinces where rice was contaminated with higher levels of cadmium due to local industry. The aim was to determine the threshold level of cadmium in urine for renal dysfunction. The microproteins β2MG (Ke et al. 2015 a) and NAG (Ke et al. 2015 b) served as effect markers. The BMDL10 values determined for β2MG were 2.00 µg cadmium/g creatinine in men and 1.69 µg cadmium/g creatinine in women (Ke et al. 2015 a). The BMDL5 for NAG was 2.08 µg cadmium/g creatinine in men and 1.93 µg cadmium/g creatinine in women (Ke et al. 2015 b). The authors concluded that the BMDLs in these Chinese populations were significantly lower than the WHO threshold of 5 µg/g creatinine for cadmium-related renal effects. The slightly lower BMDLs in women would indicate greater sensitivity to cadmium than in men. The publications do not contain any information on smoking habits or comorbidities such as diabetes.

In addition, there are several environmental health studies that are not reliable for the evaluation of a BLW (including Robles-Osorio et al. 2017; Satarug et al. 2018).

Environmental health studies are of limited use for deriving a BLW for the working population, as factors such as age, creatinine levels and diseases, including kidney disease and diabetes mellitus, are difficult to control and are of lesser importance when deriving occupational health limits.

### Derivation of a BLW for cadmium in urine

In 2008, it was stated that occupational exposure to cadmium above a concentration of 5 µg cadmium/g creatinine in urine can cause tubular kidney damage (Käfferlein et al. 2016). Studies published since 2010 on workers exposed to cadmium confirm this value in their overall assessment. Even though the study by Ding et al. (2011), which did not differentiate between smoking habits, indicates a NOAEL/LOAEL transition for tubular proteinuria in persons occupationally exposed to cadmium at approximately 3 µg cadmium/g creatinine in urine, the study by Chaumont et al. (2011) shows a BMDL5 of 5.5 to 6.6 µg cadmium/g creatinine for tubular proteinuria for never-smokers and a lower BMDL5 for ever smokers.

**Therefore, a BLW of 2 µg cadmium/g creatinine is set.**

In steady state, there are no restrictions regarding sampling time.

## Interpretation

The internationally standardised creatinine adjustment allows for comparability between the BLW and the study data without conversion. Only in elderly people with reduced muscle mass and in children and adolescents in the growth phase is the creatinine reference disadvantageous compared to the volume reference; this does not apply to employees of working age.

Since 2011, the BLW for cadmium in urine has been withdrawn. This was justified by references to the irreversibility of cadmium-induced tubular proteinuria, nephrotoxic effects below the BLW in environmental epidemiological studies, and possible glomerular kidney damage caused by cadmium. These assumptions could not be scientifically proven with certainty. Byber et al. (2015) in particular were able to refute the progression from cadmium-induced tubular to glomerular damage in their systematic review. Rather, primary glomerular or interstitial kidney disease, e.g. diabetic nephropathy or glomerulonephritis, leads to increased excretion of microproteins; not only metallothionein but also other proteins bind cadmium. Environmental health studies, especially population-based epidemiological studies, often include older people with kidney-related comorbidities; in addition, such individuals have reduced creatinine excretion due to a lack of muscle mass. These phenomena distort the statistical association between cadmium concentration in urine and microprotein excretion (known as reverse causation). Furthermore, the smoking status is rarely recorded in environmental epidemiological studies. If such confounders are not taken into account, environmental health studies are not suitable for deriving a BLW and the effects sometimes reported at low cadmium concentrations are not transferable. Unlike the study by Järup et al. (1995), the studies by Roels et al. (1997), Trzcinka-Ochocka et al. (2002) and Gao et al. (2016) suggest that tubular proteinuria is reversible after the end of cadmium exposure.

Cadmium and its inorganic compounds have been classified as confirmed human carcinogens. This is mainly due to the local effect of cadmium-containing dust on the respiratory tract. Compliance with the BLW for cadmium in urine provides protection only against the effects of the substance on the kidneys. If the BLW is exceeded, it must be checked in each individual case whether there is a kidney disease (e. g. diabetes mellitus) that leads to a reduced glomerular filtration rate (GFR) or increased glomerular proteinuria. In such cases, the cadmium level in blood should be determined; the smoking status must also be taken into account.
